# Estimating accelerated biological ageing using machine learning and metabolomics data in people with mental disorders

**DOI:** 10.1192/j.eurpsy.2023.291

**Published:** 2023-07-19

**Authors:** J. Mutz, C. M. Lewis

**Affiliations:** King’s College London, London, United Kingdom

## Abstract

**Introduction:**

Accelerated biological ageing might contribute to the higher prevalence of age-related diseases and excess mortality amongst individuals with mental disorders. Recent advances in machine learning and the collection of high-dimensional molecular “omics” data allow for the quantification of biological age.

**Objectives:**

The aim of this study was to use machine learning methods to predict biological age from nuclear magnetic resonance spectroscopy metabolomics data and to identify psychiatric traits associated with accelerated biological ageing.

**Methods:**

The UK Biobank is a multicentre community-based observational study that recruited >500,000 middle-aged and older adults. 168 metabolomic measures were quantified using the Nightingale Health platform. Phase 1 release of these data included a random subset of 118,462 UK Biobank participants. Metabolomic age delta (MetaboAgeΔ) was defined as the difference between predicted biological age and observed chronological age. We estimated group differences in MetaboAgeΔ between individuals with and without mental disorders and examined whether polygenic scores for mental disorders predicted MetaboAgeΔ.

**Results:**

Up to 110,780 participants with complete data on all metabolomic measures were included in the analysis. Individuals with a history of mental disorders had higher MetaboAgeΔ values than people without a mental illness. For example, MetaboAgeΔ suggested that the difference between predicted biological age and observed chronological age was about two-years greater amongst individuals with bipolar disorder than amongst people without mental illness. Polygenic scores for mental disorders were positively correlated with MetaboAgeΔ.

**Conclusions:**

These findings suggest that individuals with a history of mental disorders or with higher polygenic scores for mental disorders were biologically older than their chronological age.

**Disclosure of Interest:**

None Declared

